# The Harmlessness of Cervical Lacerations

**Published:** 1888-10

**Authors:** 


THE HARMLESSNESS OF CERVICAL LACERATIONS.—Dr. Emil Noeggerath has attacked an article of the gynecologists' creed in the following radical utterances: (1) Women with uterine disease conceive more easily if the cervix is lacerated than if it is intact. They abort less often in the first condition than in the second. (2) The position of the uterus is not influenced by cervical laceration. (3) The uterine axis is not lengthened by cervical laceration. (4) Erosions and ulcerations are equally frequent in lacerated and in intact cervices. (5) Erosions of the lips are never the direct result of cervical laceration. (6) Disease of the tissues of the cervix are not more frequent in lacerated than in uninjured cervices. (7) Cervical tears have no influence on the development of uterine disease either as to intensity or frequency. In his concluding remarks, he recommends that lacerations and tears be left alone.—*Boston Medical and Surgical Journal.*

